# Energy transfer across magnetopause under dawn–dusk IMFs

**DOI:** 10.1038/s41598-023-34082-2

**Published:** 2023-05-07

**Authors:** H. X. Zhang, J. Y. Lu, M. Wang

**Affiliations:** Institute of Space Weather, School of Atmospheric Physics, Nanjing University of Information Science and Technology, Nanjing, 210044 China

**Keywords:** Space physics, Magnetospheric physics

## Abstract

A parametric study on the energy transfer of the solar wind across the magnetopause entering the magnetosphere is conducted using a global magnetohydrodynamic numerical simulation. The characteristics of the mechanical and electromagnetic energy distribution under the dawn–dusk interplanetary magnetic fields (IMFs) are investigated by analyzing magnetic reconnection and viscous effect, and compared with the radial and north–south IMFs. It is shown that (1) the interactions at the magnetopause and the transfer of energy across this boundary move in relation to the IMF orientation. (2) For the duskward IMF, the mechanical energy flow clearly enters the equatorial and low-latitude regions on the dayside, and the electromagnetic energy flow has a small inflow on the equatorial and low latitudes of the dayside. A significant energy inflow appears on the dawn side in the northern hemisphere and the dusk side in the southern hemisphere near the polar cusp. (3) The energy distribution characteristics across the magnetopause under dawn–dusk IMFs are mirror symmetric about the $Y=0$ plane. (4) For a magnetic field of 5 nT, the electromagnetic energy input under the dawn–dusk IMFs is twice as large as the mechanical energy and the electromagnetic energy under the radial IMF, which is five times as large as the electromagnetic energy during the pure northward IMF, but only half as large as the electromagnetic energy under the pure southward IMF. The mechanical energy input under dawn–dusk IMFs has the same magnitude as that under radial and north–south IMFs. The magnitude of the energy transfer rate for the dawn IMF and dusk IMF (about 3.5%) is between 1.71% for the northward IMF and 4.95% for the southward IMF, but higher than 2.22% for the radial IMF. The Akasofu-type energy-coupling formula, $\varepsilon$, underestimates the energy input from the solar wind under $B_{y}$ dominated IMF.

## Introduction

The magnetic reconnection between the interplanetary magnetic field (IMF) and geomagnetic field is the most critical process that controls the energy transfer from the solar wind to the magnetosphere across the magnetopause. Compared as the geomagnetic field is relatively stable, the variable IMF as a part involved in the interaction naturally plays a key role in this coupling system. The reconnection can occur for nearly any orientation of the IMF and produce different reconnection rates under different IMF directions. The reconnection efficiency affects the amount of energy input^1^, therefore, understanding the energy distribution characteristics, the amount of energy input, and energy transfer efficiency under different directions of IMF is worth exploring.

The effect of the north–south components of IMF, $B_z$, on the magnetopause magnetic reconnection of the magnetosphere is the most pronounced, and many works have investigated the magnetic reconnection and the energy transport under the northward or southward IMF^2–4^. It was demonstrated that the reconnection for southward IMF always occurs near the equatorial plane on the magnetopause, where the reconnection rate is the greatest. In contrast, if the IMF is northward, the magnetic reconnection always occurs near the tail behind the polar cusp, and the reconnection becomes the weakest^5^. Using the polar cap potential difference as an equivalent proxy for the magnetic reconnection rate, the reconnection rate under northward IMF is about 10–30% of that under southward IMF^6^.

The impact of the radial component of the IMF on magnetopause^7^, magnetic reconnection, and energy transfer across the magnetopause was investigated recently. A radial IMF can make the reconnection location and energy transfer asymmetric in the northern and southern hemispheres^8,9^. Petrinec et al.^10^ found that the reconnection site on the magnetopause during radial IMF (without considering the strong radial IMF) is not exactly at the theoretically estimated position but is located further downstream of the magnetopause. Using a global magnetosphere MHD model combined with the THEMIS observations, Pi et al.^11^ studied the global structure of the adjoining regions near the magnetopause under radial IMF and showed that there is a hemispheric asymmetry of the reconnection location of the dayside magnetopause. For a non-null Earthward IMF $B_{x}$, the magnetic reconnection occurs on the dayside magnetopause in the southern hemisphere and behind the cusp in the northern hemisphere. Similarly, using a global MHD simulation, Peng et al.^12^ found that the dayside reconnection line of the magnetopause moves northward (southward) when the $B_{x}\geqslant$ 0 ($B_{x}\leqslant$ 0) , resulting in the magnetopause movement. Hoilijoki et al.^13^ used another global MHD simulation to find that the radial IMF mainly affects the reconnection position on the dayside magnetopause and has a more significant impact on the intensity of energy transfer in the load zone (the region where energy is converted from magnetic to kinetic form, such as the subsolar point of the magnetopause where reconnection occurs.) Lu et al.^14^ used a global MHD model to study the energy transfer across the magnetopause under radial IMF and found that the energy transfer across the magnetopause is asymmetric and can not be estimated by the Akasofu-type energy-coupling formula. The formula is $\varepsilon =\frac{4\pi }{\mu _{0}}vB^{2}L^{2}_{0}\sin ^{4}\frac{\theta }{2}$, where *v* is the solar wind velocity, *B* is the IMF intensity, $L_0$ = 7 $R_E$ is an empirical scaling parameter, and $\theta$ is the IMF clock angle ($\tan {\theta } = \frac{B_{y}}{B_{z}}$). Particularly, the electromagnetic energy and the energy transfer rate of radial IMFs is lower than the one of pure southward IMF but higher than the one of pure northward IMF.

The dawn–dusk component of the IMF also affects magnetopause and magnetic reconnection^15,16^. When the Y component of the IMF dominates, the interplanetary magnetic field is reconnected with the geomagnetic field on the day side. The open magnetic field lines connected to the Earth move to the magnetotail and reconnect with the geomagnetic field near the tail lobe of the magnetosphere again, forming the low latitude boundary layer^17,18^. Marcucci et al.^19^ found that the direction of the accelerated plasma flow changes when the IMF turns from dusk to dawn, as evidence of a change in the reconnection position at the magnetopause. Park et al.^20^ calculated the electric field at the magnetopause and found that anti-parallel reconnection is more important than the component reconnection for the case of slight dipole tilt and IMF $B_{y}$ component. Komar et al.^21^ tested and compared a number of existing models predicting the location of magnetic reconnection at dayside magnetopause for various solar wind conditions. They found that the maximum magnetic shear model does not rotate with the magnetic separators for different IMF clock angles in the simulation without dipole tilt. Cowley et al.^22^ discussed the observed magnetospheric asymmetry caused by IMF $B_{y}$ according to an open model of the magnetosphere. In addition, the dawn–dusk IMFs can also lead to the asymmetric distribution of the field aligned current (FACs) in the reconnection region of the magnetopause and increase the FACs in the reconnection region^23^. Using the Cluster data, Cheng et al.^24^ selected 748 FACs cases and proposed a significant north–south asymmetry in the polarity of the FACs under the dawn–dusk IMFs, especially in the duskward IMF. A north–south asymmetry induced by the asymmetry of the tail lobe pressure occurs in the magnetosphere-ionosphere system when there is a significant IMF $B_{y}$ component, and the asymmetry appears during low tail reconnection and decreases during enhanced tail reconnection^25^. When the Earth’s dipole is tilted in the direction corresponding to northern winter, the area of the polar cap under duskward IMF caused by the difference of the day-side reconnection rate is larger than that of dawnward IMF^26^. Cao et al.^27^ proposed that the penetration with IMF $B_{y}$ into the magnetosphere is enhanced during strong magnetospheric convection. Guo et al.^28^ used a global MHD simulation to investigate the effects of IMF $B_{y}$ on the FAC closure in the magnetosphere. The symmetry of the FACs across the noon-night meridional plane breaks in the ionosphere for dawn–dusk IMFs, unlike those cases in which the IMF is purely northward or southward. Using satellite observations and a global MHD model coupled with a ring current model, Holappa et al.^29^ found that in the northern hemisphere, the flux of energetic magnetospheric protons and the growth rate of the circulation are larger for IMF $B_{y}\leqslant$ 0 than for IMF $B_{y}\geqslant$ 0. The above studies show that the magnetospheric structure and magnetic reconnection are affected by the IMF $B_{y}$ component, and the energy transfer characteristics across the magnetopause are also impacted. However, few quantitative studies on the distribution characteristics of energy transmission across the magnetopause and the energy transport rate have been reported under the dawn-dusk IMFs.

Another important factor contributing to mass and momentum transport across the magnetopause is the presence of viscous interactions between the solar wind and the magnetosphere that produce the magnetospheric convection^30^. It has been reported that the low latitude magnetopause is more conducive to the generation of K-H instability and viscous interaction when the IMF is northward^31^ and the magnetotail viscosity under radial IMF is weaker than that in northward IMF^14^. However, the effect of viscous interactions under IMF $B_{y}$ is still unclear.

In this paper, through the global MHD simulations, we split the energy into mechanical energy and electromagnetic energy, and discuss their distribution on the magnetopause under $B_{y}$ dominated IMF, respectively. The energy transfer mechanism under dawn–dusk IMFs is also given by analyzing the location of magnetic reconnection occurrence and viscous effect. In addition, the energy transport rates under IMF $B_{y}$ are compared with the one obtained for the radial and north–south IMFs.

## Global MHD model and methods

We use the Space Weather Modeling Framework (SWMF), a well-established set of computational models developed by the University of Michigan, to simulate the physical processes between the Sun and the Earth. SWMF is widely used to study the various effects of solar wind on the magnetosphere, such as the convection in the northward IMF^32–34^, the IMF $B_{y}$^35,36^, dipole tilt angle^37^, and Parker-spiral IMF conditions^38^ on the magnetosphere. The SWMF numerical model, including the fully coupled RCM module, is also used to study the influence of the southward component of the IMF^39^, the dynamics of magnetic storms^40^, and the energy transfer across the magnetopause^2,41^. The methods of our research are similar to the one we used to study the energy transmission across the magnetopause for purely southward and purely northward IMF^2,14^.

In a typical simulation used in this work, the computational domain is defined by − 224 $R_{E}\leqslant$ X $\leqslant$ 32 $R_{E}$ and − 64 $R_{E}\leqslant$ Y, Z $\leqslant$ 64 $R_{E}$ in GSM coordinates, with a grid size of 4 $R_{E}$. For − 64 $R_{E}\leqslant$ X $\leqslant$ 32 $R_{E}$ and − 64 $R_{E}\leqslant$ Y, Z $\leqslant$ 64 $R_{E}$, the grid size is 2 $R_{E}$; inside − 48 $R_{E}\leqslant$ X $\leqslant$ 32 $R_{E}$ and − 32 $R_{E}\leqslant$ Y, Z $\leqslant$ 32 $R_{E}$, the grid size is 1 $R_{E}$; inside − 32 $R_{E}\leqslant$ X $\leqslant$ 24 $R_{E}$ and − 16 $R_{E}\leqslant$ Y, Z $\leqslant$ 16 $R_{E}$, the grid size is $\frac{1}{2}R_{E}$; inside − 28 $R_{E}\leqslant$ X $\leqslant$ 16 $R_{E}$ and − 12 $R_{E}\leqslant$ Y, Z $\leqslant$ 12 $R_{E}$, the grid size is $\frac{1}{4}R_{E}$; and inside − 14 $R_{E}\leqslant$ X $\leqslant$ 10 $R_{E}$ and − 8 $R_{E}\leqslant$ Y, Z $\leqslant$ 8 $R_{E}$, the grid size is $\frac{1}{8}R_{E}$. The inner boundary is a sphere with a radius of 3 $R_{E}$.

To compare and discuss the influence of each of the IMF components on the energy transmission, we first analyze the influence of the variable IMF $B_{y}$ on the energy transmission across the magnetopause, a case that has not been studied before. As shown in Table 1, the solar wind conditions used in our study in cases 1–7 only change the strength and direction of IMF $B_{y}$. In cases 8–11, we only changed the direction of IMF $B_{x}$ and $B_{z}$, with a magnetic field strength of 5nT. The dipole tilt of all conditions is set to zero, and other physical quantities remain consistent.

This work investigates energy transfer under a steady solar-wind condition and IMF. As Kelvin-Helmholtz instabilities are not considered here, magnetic reconnection is then the major contributor to the coupling. Since we are dealing with the large-scale coupling of solar wind and magnetosphere, we did not make special set with resistivity.

**Table 1:** Parameters for synthetic simulations.

*RunNO*.	$B_{x} [nT]$	$B_{y} [nT]$	$B_{z} [nT]$	$P_{d} [nPa]$	$\mathbf {v_x} [km/s]$	*n*[/$cm^{-3}]$	$\mathbf {v_{y, z}}[km$/*s*], $tilt[^\circ ]$
Case 1	0	1	0	3	500	7.2	0
Case 2	0	5	0	3	500	7.2	0
Case 3	0	10	0	3	500	7.2	0
Case 4	0	15	0	3	500	7.2	0
Case 5	0	20	0	3	500	7.2	0
Case 6	0	25	0	3	500	7.2	0
Case 7	0	− 5	0	3	500	7.2	0
Case 8	5	0	0	3	500	7.2	0
Case 9	− 5	0	0	3	500	7.2	0
Case 10	0	0	5	3	500	7.2	0
Case 11	0	0	− 5	3	500	7.2	0

For each case, SWMF is run for two hours to get a stable magnetosphere. Then, we adopt the method of tracking the solar wind streamline to automatically identify the smooth surface of the magnetopause and the approach of calculating the pressure gradient to determine the location of the polar cusp as done in previous studies^2,14,34^. The intersection line between the posterior edge of the cusp (located at the maximum value of the pressure gradient) is located, and the identified magnetopause is defined as a boundary to separate the dayside and nightside magnetopause. Once the coordinates of the three-dimensional magnetopause surface are obtained, the transfer energy can be calculated^2,14,42^. The total energy input to the magnetosphere is defined as:

1 $$\begin{aligned} W_{g} = W_{m}+W_{p} = \int \;d{W_{m}} + \int \,d{W_{p}} = \int \;dS(\mathbf {K_{m}}+\mathbf {K_{p}}) \cdot \vec {\textbf{n}} \end{aligned}$$

where $W_{m}$ is the total mechanical energy input, $W_{p}$ is the total electromagnetic energy input, *dS* is the area of the surface element, and $\vec {\textbf{n}}$ is the unit vector perpendicular to the surface pointing outward the magnetopause. $\mathbf {K_{m}}$ represents the mechanical energy flux density, which is oriented in the same direction as the magnetosheath flow field, and $\mathbf {K_{p}}$ represents the electromagnetic energy flux whose direction is determined by the direction of $\textbf{E} \times \textbf{B}$. The definition formulas of $\mathbf {K_{m}}$ and $\mathbf {K_{p}}$ are

2 $$\begin{aligned} \mathbf {K_{m}} = (U + P - \frac{B^{2}}{2\mu _{0}})\vec {\textbf{v}} \end{aligned}$$

3 $$\begin{aligned} \mathbf {K_{p}} = \frac{1}{\mu _{0}} \textbf{E} \times \textbf{B} \end{aligned}$$

where *P* is the pressure, $\textbf{B}$ is the magnetic field, $\textbf{v}$ is magnetosheath velocity, $\textbf{E} = \textbf{B} \times \textbf{v}$ is the electric field, and the total energy density of the fluid element *U* is defined as $U = \frac{P}{\gamma -1} + \frac{\rho v^{2}}{2} + \frac{B^{2}}{2\mu _{0}}$ ($\gamma = \frac{5}{3}$).

Using the above equations, the mechanical energy and electromagnetic energy injected into the magnetosphere are calculated for all azimuths (the angle away from the positive Y-axis in the *YZ* plane) from the subsolar point to $X=-40 R_{E}$ of the magnetotail in the *YZ* plane. When energy is injected into the magnetosphere, the energy value is negative, and vice versa.

## Energy flow through magnetopause for dawn–dusk IMFs

Since the characteristics of energy transfer under the condition of $B_{y}= -5$ nT and $B_{y}= 5$ nT with $P_{d}= 3$ nPa have an approximately mirror symmetry, here we analyze the mechanism of energy transfer under the duskward IMF as an example.

**Figure 1:**
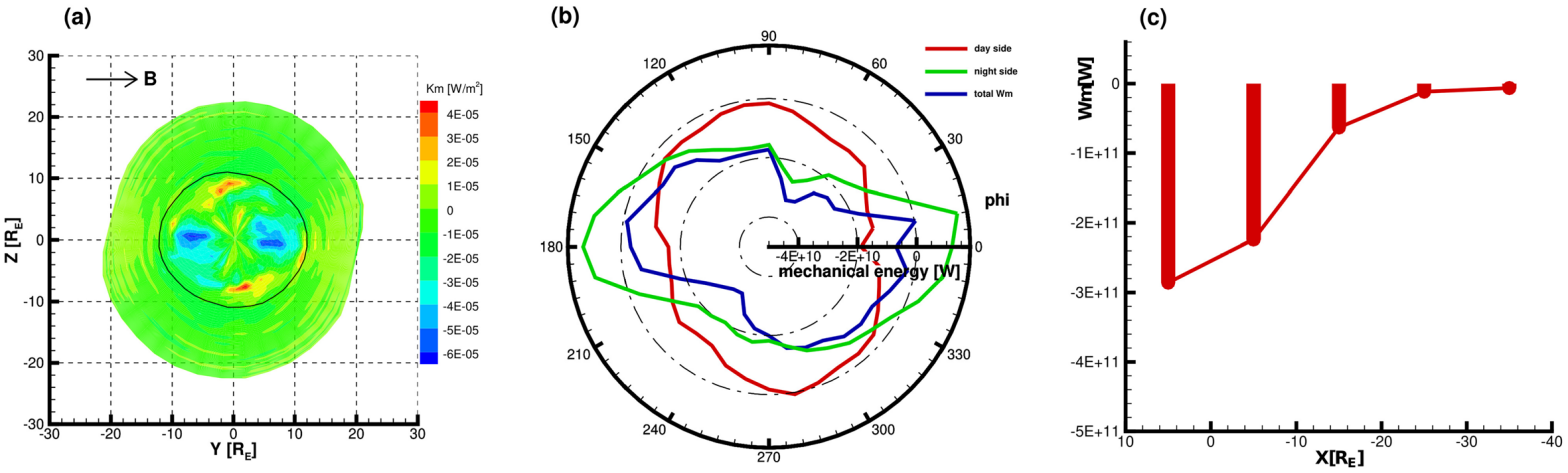
The energy transfer for $B_{y}= 5$ nT and $P_{d}= 3$ nPa: (**a**) mechanical energy flux across the magnetopause in a projection on the YZ plane viewed from the sunward direction. Blue (red) color indicates inward (outward) energy at the magnetopause. The solid black line represents the intersection of the magnetopause with the plane $X = X_{cusp}$ (tailward edge of the cusp region); (**b**) the distribution of mechanical energy with azimuth for the dayside magnetopause (red line), the nightside magnetopause (green line), and the entire magnetopause (blue line), the negative (positive) values represent inward (outward) energy; (**c**) mechanical energy transfer across the magnetopause integrated along the X-axis over the following X coordinate intervals: X $>X_{cusp}$, $-10 R_{E}<$ X $\leqslant X_{cusp}$, $-20 R_{E}<$ X $\leqslant -10 R_{E}$, $-30 R_{E}<$ X $\leqslant -20 R_{E}$, and $-40 R_{E}<$ X $\leqslant -30 R_{E}$.

**Figure 2:**
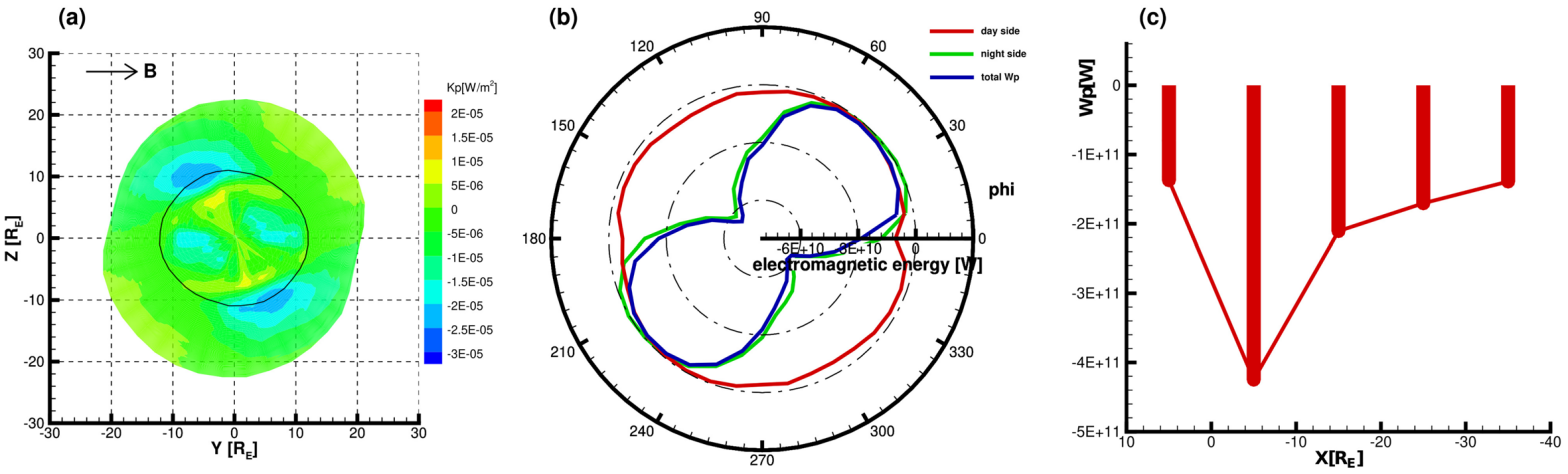
Same as Fig. 1 but for electromagnetic energy transfer.

Figure 1a shows the mechanical energy density flux projected on the *YZ* plane across the magnetopause as viewed from the sunward direction for $B_{y}= 5$ nT and $P_{d}= 3$ nPa. Flowing into the magnetosphere is negative, shown in blue, while the red region indicates the energy flowing out of the magnetosphere. The solid black line represents the boundary where the polar cusp region is located in the plane $X = X_{cusp}$ identified by the pressure gradient and the surface of the magnetopause intersect. Thus the region inside the solid black line represents the dayside magnetopause, and the outer side represents the nightside. Figure 1b shows the distribution of mechanical energy with azimuth angle for the dayside magnetopause (red line), the nightside magnetopause (green line), and the entire magnetopause (blue line), respectively. Figure 1c shows the distribution of the energy integrals of the mechanical energy transfer within the following X-axis intervals: X $>X_{cusp}$, − 10 $R_{E}<$ X $\leqslant X_{cusp}$, − 20 $R_{E}<$ X $\leqslant$ − 10 $R_{E}$, − 30 $R_{E}<$ X $\leqslant$ − 20 $R_{E}$, and − 40 $R_{E}<$ X $\leqslant$ − 30 $R_{E}$. Figure 2 is the same as Fig. 1 but for electromagnetic energy transfer.

It can be seen from Figs. 1a and 2a that the symmetry axes of the magnetopause shape rotate clockwise relative to the meridian plane when only the Y component of the IMF exists. When the dipole tilt is zero, the point of the maximum distance between the magnetopause projected onto the *YZ* plane and the origin is on the z-axis under the north–south and radial IMFs. However, in the dawn–dusk IMFs, the line between this point and the origin deflects the z-axis by $18^{\circ }$ approximately. For the mechanical energy transfer, on the day side of the magnetopause, one can see a mechanical energy flux inflow near the equator and low latitude, but a small area and magnitude of mechanical energy outflow at the high latitude in Figure 1a. On the nightside, there is a large and weak mechanical energy inflow at the middle and high latitudes in the northern and southern hemispheres, in which a small area of relatively large mechanical energy inflow occurs behind the cusps. At the same time, there is a weak outflow energy flow near the low latitude magnetotail. As shown in Figure 1b, we find that the distribution of mechanical energy with azimuth on the entire magnetopause is not symmetrical between the northern and southern hemispheres but is distorted. It is easy to see that the area with the largest mechanical energy input is near the equator on the day side. In contrast, the mechanical energy input is relatively small at a high latitude. For the night side, the maximum energy input appears near azimuth $75^{\circ }$ and $255^{\circ }$, i.e., the maximum mechanical energy input occurs on the dusk side in the northern hemisphere and the dawn side in the southern hemisphere, and the mechanical energy output is presented near the low latitude with the maximum output near the equator. In general, the energy transfer on the night side has a relatively large contribution to the mechanical energy transfer across the magnetopause. Figure 1c illustrates that the net input of mechanical energy reaches the maximum on the dayside of the magnetopause, then follows in the near-Earth magnetotail, while the input decreases significantly in the three regions of $-20 R_{E}<$ X $\leqslant -10 R_{E}$, $-30 R_{E}<$ X $\leqslant -20 R_{E}$, and $-40 R_{E}<$ X $\leqslant -30 R_{E}$.

Figure 2a shows a small area and magnitude of electromagnetic energy outflow at the front of the polar cusp, and the maximum value of the outflow energy flow is around the azimuthal angles $100^{\circ }$ and $280^{\circ }$. In addition, the rest of the positions on the dayside shows the inflow of electromagnetic energy, and the maximum value of the inflow is located near the equator. Both the inflow and outflow extremes are small and the density flux intensity is weak. However, a large area of electromagnetic energy inflow appears in the far magnetotail with a maximum value near the polar cusp, except in the mid-latitude region of the far magnetotail, where there is a small area and weak electromagnetic energy outflow. Figure 2a shows that the electromagnetic energy flux enters into the magnetosphere in the region near the equator on the dayside, which is confirmed by the distribution of the dayside electromagnetic energy integral with an azimuth in Fig.  2b. Meanwhile, as shown in Fig.  2b, the electromagnetic energy input is also present near the azimuthal angles $120^{\circ }$ and $300^{\circ }$, which is opposite to the outflow shown in Fig.  2a, because there is a small area of outflow but a more extensive area of inflow on the dayside. For the night-side magnetotail, Figure 2b illustrates that the electromagnetic energy input is the greatest in the night-side magnetotail around the azimuths of $135^{\circ }$ and $315^{\circ }$. In addition, it can be seen that the contribution of energy transfer on the nightside to the entire magnetopause is more significant. Also, the net input of electromagnetic energy is the largest in the near-Earth magnetotail ($-10 R_{E}<$ X $\leqslant X_{cusp}$), which is much larger than the maximum value of the net mechanical energy input as shown in Fig.  2c.

**Figure 3:**
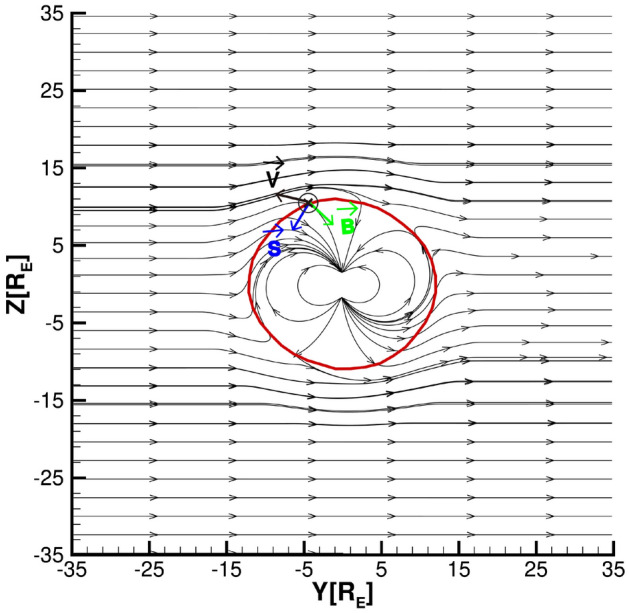
Illustration of the energy transfer for $B_{y} = 5$ nT. The red solid line represents the magnetopause in the *YZ* plane, the black arrows represent the solar-wind speed ($\textbf{v}$) direction, the green arrows represent the magnetic field ($\textbf{B}$) direction, and the blue arrows represent the Poynting vector ($\textbf{S}$) direction.

Next, we analyze the energy transfer mechanism for duskward IMF. The magnetic field line distribution at $B_{y}= 5$ nT and $P_{d}= 3$ nPa is shown in Fig.  3. The gray vector line represents the distribution of magnetic field lines in the *YZ* plane where $X = X_{cusp}$ is located, the red solid line represents the *XZ* plane projection of the magnetopause, the black arrows represent the direction of solar wind velocity on the magnetopause surface ($\textbf{v}$), the direction of magnetic field ($\textbf{B}$) is indicated by the green arrows, and the Poynting vector ($\textbf{S}$) is represented by the blue arrows. It can be seen that the reconnection occurs near the dusk side of the northern hemisphere and the dawn side of the southern hemisphere. After the reconnection occurs in both the northern and southern hemispheres, the reconnected magnetic lines are dragged towards the dawnward and duskward at the north and south poles, respectively. At the same time, due to the effect of the solar wind, the reconnected magnetic lines move tailward and eventually become open in the magnetotail. Therefore, the magnetic reconnection occurs on the dusk side of the northern hemisphere and the dawn side of the southern hemisphere, resulting in the accumulation of magnetic field lines near the dawn-north side and the dusk-south side, and the magnetic field lines move toward the magnetotail under the dragging effect of the solar wind.

During duskward IMF, the northern-dusk and the southern-dawn regions of the magnetopause are opened by magnetic reconnection. The accelerated plasma flow is then deflected from the nominal shocked solar wind flow (Fig.  3). As a result, the work of mechanical energy makes the plasma flow enter the magnetosphere from the magnetic field lines opened by magnetic reconnection, along with the magnetic field lines convection to the magnetotail, becoming a part of the plasma mantle, as shown in Fig.  1a. After the asymmetric reconnection of the northern and southern hemispheres, the IMF and the Earth’s magnetic field are no longer parallel. According to formulas $\textbf{E} = \textbf{B} \times \textbf{v}$ and $\textbf{S} = \frac{1}{\mu _{0}} \textbf{E} \times \textbf{B}$, vector $\textbf{B}$ points to the inner side of the magnetosphere, which means that the electromagnetic energy input occurs in the dawn side of the northern hemisphere and the dusk side of the southern hemisphere, as shown in Figs.  2a and 3.

**Figure 4:**
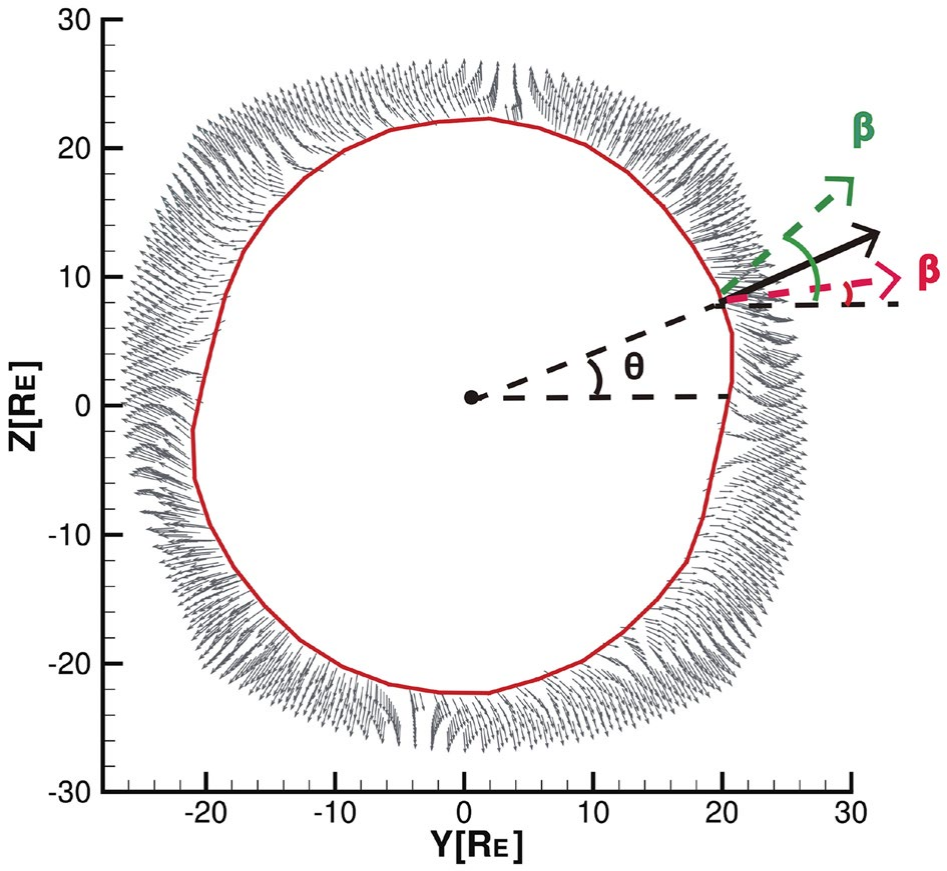
Sketch of streamline deflection (viscous effect) projected on the first quadrant of the *YZ* plane for $X = -35 R_{E}$ viewed from the sunward direction.

As reported by Lu et al.^2,14^, magnetic reconnection is not the only factor affecting the energy transfer, and viscous effect in the magnetotail also has an important role for the energy transfer across the magnetopause, where the plasma has not only the radial velocity flowing to the magnetotail but also the tangential velocity flowing along the magnetopause from high latitude to low latitude near the magnetopause. To analyze the viscous effect, the velocity vector diagrams (gray vectors) of the streamlines near the magnetopause in the *YZ* plane for $X = -35 R_{E}$ are shown in Fig.  4, where we take the region of $Y>0$ and $Z>0$ as an example, and the black arrow indicates the direction of the connection between the origin and the magnetopause in the *YZ* plane, $\theta$ is the angle between the black arrow and the positive Y-axis (azimuth angle), and $\beta$ is the angle between the velocity vector projected onto the *YZ* plane and the Y-axis. We define $\beta -\theta$ as the streamline deviation angle in the *YZ* plane. In the absence of external force, the viscous force is zero, and the plasma velocity does not deflect, and then the direction of the black arrow is the direction of the streamline. However, when the viscous force is non-zero, if $\beta > \theta$, as shown in the green line of Fig.  4, the streamline rotates counterclockwise along the magnetopause under the effect of viscosity, otherwise, the streamlines rotate clockwise along the magnetopause, as seen in the red line. The greater the absolute value of the deflection angle, the greater the degree of turning.

Figure 4 shows that the deflection direction of the streamlines in the magnetotail is different from that in the north–south and radial solar wind conditions^2,14^. Under the duskward IMF, the streamlines flow from the dusk side of the northern hemisphere (dawn side of the southern hemisphere) to the southern hemisphere dusk side (northern hemisphere dawn side), respectively. The plasma flowing into the lower latitudes accumulates in the southern hemisphere dusk side and northern hemisphere dawn side regions with increased density, in which a small amount flows into the magnetosphere at the lower latitudes, while most of the remaining flow out of the magnetopause. This explains the deflection of the plasma streams at the outflow and inflow positions of the magnetotail under the duskward IMF, as shown in Fig.  1a.

## Comparison between dawn–dusk, north–south, and radial IMFs

**Figure 5:**
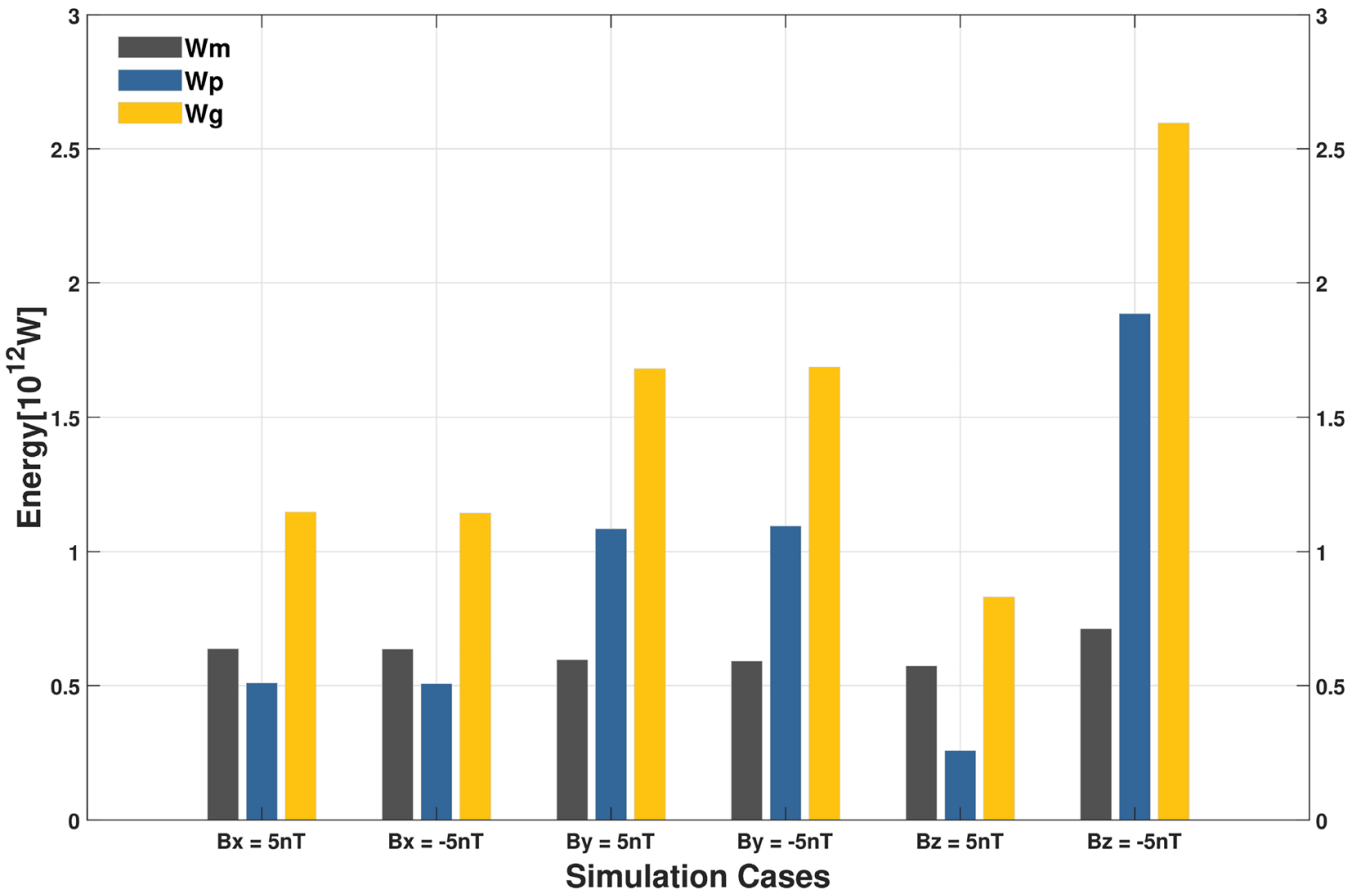
Comparison of $W_m$, $W_p$, and $W_g$ of six simulation cases (sunward, earthward, duskward, dawnward, northward, and southward IMF, respectively). The magnitudes of the magnetic field and dynamic pressure are 5 nT and 3 nPa, respectively.

The magnetopause energy transfer in the the case of radial, the dawn-dusk, and the north–south IMFs are compared. The mechanical energy ($W_{m}$), electromagnetic energy ($W_{p}$), total energy injected into the magnetosphere ($W_{g}$), total energy of solar wind impacting the magnetopause ($W_{SW}$), and energy transmission rate are calculated and compared for the conditions listed in Table 2. As the strength of the magnetic field for increasing duskward IMFs, there is a significant increase in electromagnetic energy and total energy input, while only a tiny increase in mechanical energy. Moreover, the total energy of the solar wind energy impinging on the magnetopause appears to decrease significantly because the surface area of the magnetopause decreases due to the enhanced magnetic field. Figure 5 compares the results of $W_{m}$, $W_{p}$, and $W_{g}$ for six simulation cases (radial IMF, dusk-dawn IMFs, and north–south IMFs, respectively), we find that the electromagnetic energy is about twice the mechanical energy input for the dusk-dawn IMFs. For mechanical energy, the inputs are comparable for the radial, dusk-dawn, and north–south IMFs, while for electromagnetic energy, the input in the dusk-dawn IMFs is twice that in the radial IMF, four times that in the northward IMF, but only half that of the southward IMF. The energy transfer rate is then calculated from the total energy $W_g$ injected into the magnetosphere and the solar wind energy $W_{SW}$ impinging on the magnetopause, given in the last column in Table 2. The energy transfer rates of 3.58% for dawnward IMF and 3.52% for duskward IMF are higher than 2.22% in the radial IMFs and 1.71% during northward IMF, but lower than 4.95% during southward IMF.

**Figure 6:**
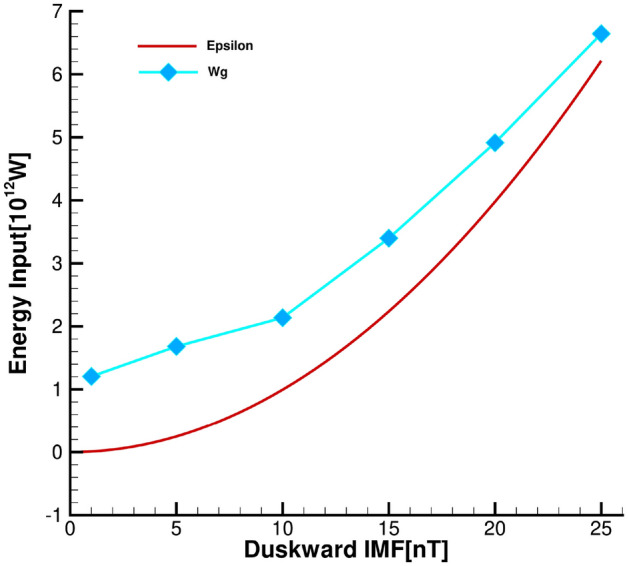
The energy-coupling formula $\varepsilon$ and $W_g$ as a function of duskward IMF for $P_{d}= 3$ nPa.

Figure 6 shows the energy input as a function of duskward IMF for the simulations with $P_{d} = 3 nPa$. The red line represents the $\varepsilon$ parameter, an energy coupling function proposed by Akasofu et al.^43^, and the light blue line represents $W_{g}$. It can be seen that the total energy injected into the magnetosphere under dawn–dusk IMFs follows the same trend as the energy coupling function proposed by Akasofu et al.^43^, similar to the trend in the north–south condition^2^, but different from the radial condition^14^. However, $\varepsilon$ obviously underestimates the energy injected into the magnetosphere. The difference between the formula and the result may be due to the identified magnetopause in this work is an open surface and the plane at the magnetotail is not considered (the application of the numerical simulation results does not allow to consider the energy transfer in the plane of the magnetotail for the time being). The formula represents the energy dissipated in the magnetosphere and ionosphere, which means that not all of it enters the inner magnetosphere, but a portion flows out of the magnetotail. The outflow from the magnetotail is not considered in this work.

**Table 2:** The mechanical energy ($W_m$), electromagnetic energy ($W_p$), the total energy ($W_g$) through the entire magnetopause, and the solar wind energy ($W_{SW}$) impinging on the magnetopause in the cases of Table1.

Run NO	SW parameters	$W_m[10^{12}W]$	$W_p[10^{12}W]$	$W_g[10^{12}W]$	$W_{SW}[10^{12}W]$	Transfer rate $(\%)$
Case 1	$B_y$= 1nT, $P_d$= 3nPa	0.589	0.616	1.205	51.228	2.35
Case 2	$B_y$= 5nT, $P_d$= 3nPa	0.596	1.085	1.681	47.766	3.52
Case 3	$B_y$= 10nT, $P_d$= 3nPa	0.656	2.137	2.792	41.226	6.77
Case 4	$B_y$= 15nT, $P_d$= 3nPa	0.708	3.399	4.106	37.417	10.97
Case 5	$B_y$= 20nT, $P_d$= 3nPa	0.789	4.914	5.704	34.981	16.31
Case 6	$B_y$= 25nT, $P_d$= 3nPa	0.851	6.643	7.493	33.244	22.54
Case 7	$B_y$= − 5nT, $P_d$= 3nPa	0.591	1.095	1.686	47.120	3.58
Case 8	$B_x$= 5nT, $P_d$= 3nPa	0.637	0.510	1.147	51.592	2.22
Case 9	$B_x$= − 5nT, $P_d$= 3nPa	0.636	0.507	1.143	51.612	2.22
Case 10	$B_z$= 5nT, $P_d$= 3 nPa	0.573	0.258	0.831	48.477	1.71
Case 11	$B_z$= − 5nT, $P_d$= 3 nPa	0.711	1.885	2.596	52.436	4.95

**Figure 7:**
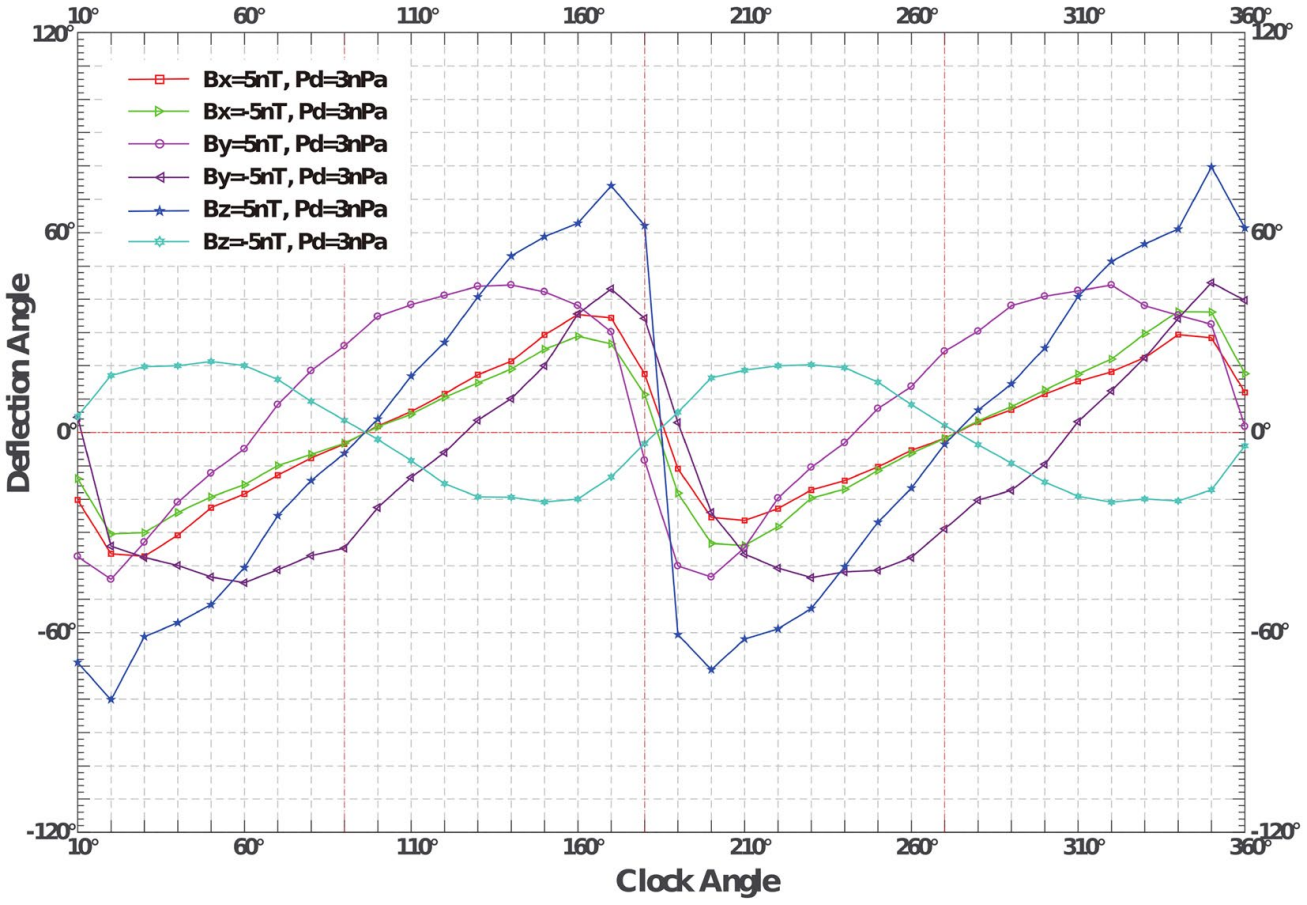
Deflection angle ($\beta -\theta$) distribution with deviation angle. When the deflection angle exceeds zero, the streamline deflects counterclockwise and vice versa.

In addition, we also compare the deflection degree of the magnetotail streamlines for the dawn-dusk, the radial, and the north–south IMFs (|B|=5nT) under the dynamic pressure is 3nPa, the velocity is 500 *km*/*s*, and the density is 7.2 $cm^{-3}$, as shown in Fig.  7. Lu et al.^14^ have already mentioned in detail that the viscous effect in radial IMF is weaker than in northward IMF. It can also be seen from Fig.  7 that the absolute values of the maximum streamline deflection angle in the magnetotail under the dawn and dusk IMFs are equivalent, and larger than under the radial IMF, but smaller than in the northward IMF.

## Summary and conclusions

In summary, the interaction between the solar wind and magnetosphere is investigated by using a three-dimensional adaptive MHD model. The magnetopause surface is identified by the streamline method, and the total energy across the magnetopause under dawn–dusk IMFs is divided into mechanical energy and electromagnetic energy. Magnetic reconnection and viscous interaction processes are used to understand the physical mechanism of energy transfer from the solar wind to the magnetosphere. The main conclusions of this paper are as follows:

1. The symmetry axes of the magnetopause rotate clockwise relative to the meridian plane when only the Y component of the IMF exists. The line between the point on the magnetopause projected on the *YZ* plane farthest from the origin and the origin deviates from the z-axis, about $18^{\circ }$ under the considered condition in the paper.
2. For the duskward IMF, the energy transfer distribution is no longer symmetric in the north–south direction when viewing from the sunward direction, the mechanical energy flow enters the equatorial and low-latitude regions on the dayside. The electromagnetic energy has a small magnitude of inflow at the equator and low latitudes of the dayside, and a significant energy inflow occurs on the dawn side of the northern hemisphere and the dusk side of the southern hemisphere near the polar cusp. The characteristics of energy distribution across the magnetopause under dawnward and duskward IMF are mirror symmetric about the $Y=0$ plane.
3. For the same IMF strength (5nT here), the electromagnetic energy input under the $B_{y}$ oriented IMF is twice as large as the mechanical energy under the IMF $B_{y}$ and the electromagnetic energy under the radial IMF, which is five times as large as the electromagnetic energy during the northward IMF, but only half as large as the electromagnetic energy under the southward IMF. The mechanical energy input under dawn–dusk IMFs is the same order of magnitude as that under radial and north–south IMF.
4. For solar wind condition we considered, the energy transfer rates of 3.58% for dawnward IMF and 3.52% for duskward IMF are higher than 2.22% in the radial IMFs and 1.71% during northward IMF but lower than 4.95% during southward IMF.
5. With increasing IMF, the change of mechanical energy input is insignificant for the dawn–dusk IMFs, while the electromagnetic energy input increases nonlinearly. The Akasofu-type energy-coupling formula, $\varepsilon$, obviously underestimates the energy input from solar wind to magnetosphere for the cases dominated by IMF $B_{y}$.

## Data Availability

The data comes from the simulation for the steady states under different cases, which depends only on the solar wind conditions for fixed ionospheric conductivities. The datasets used and/or analyzed in this study are available from the corresponding author on request by readers.
